# A novel species of bothriocephalid cestode, *Clestobothrium brettiae* n. sp. in the European hake (*Merluccius merluccius*) revealed using an integrative taxonomic approach

**DOI:** 10.1017/S0031182025100887

**Published:** 2025-10

**Authors:** Flavia Occhibove, Alejandro López-Verdejo, Mario Santoro

**Affiliations:** 1 Department of Integrative Marine Ecology, Stazione Zoologica Anton Dohrn, Naples, Italy; 2 Marine Zoology Unit, Cavanilles Institute of Biodiversity and Evolutionary Biology, University of Valencia, Paterna, Spain; 3 NBFC, National Biodiversity Future Center, Palermo, Italy

**Keywords:** Bothriocephalidae, Eastern Mediterranean Sea, fish tapeworm, integrative taxonomy, molecular analysis, multivariate analysis, new species, phylogeny

## Abstract

*Clestobothrium* Lühe, 1899 is a genus of cestodes belonging to the order Bothriocephalidea, which infects marine fish from the Gadiformes order. Herein, a novel species of *Clestobothrium* is described from the intestine of the European hake *Merluccius merluccius* (Linnaeus, 1758) in the Ionian Sea (Eastern Mediterranean Sea), through an integrative taxonomic approach that combines morphological and molecular data. The new species, named *Clestobothrium brettiae* n. sp., can be distinguished from all congeners by its unique ovary shape, and a combination of characters including the arrangement and number of testes. It shares similar morphological characters, with *Clestobothrium crassiceps* (Rudolphi, 1819) Lühe, 1899, which overlaps in host and geographic distribution. However, morphological differences between *Clestobothrium brettiae* n. sp. and *C. crassiceps* also include the arrangement of gladiate spinitriches, as well as a larger scolex, proglottids, testes and cirrus sac, and different ovary morphology. The establishment of *Clestobothrium brettiae* n. sp. as a novel taxon is supported by detailed morphological description and biometric statistics, in addition to molecular characterisation (based on partial small subunit ribosomal ribonucleic acid [rRNA], partial large subunit rRNA, and internal transcribed spacer region 2), genetic distance, and phylogenetic analyses.

## Graphical abstract

**Figure:**
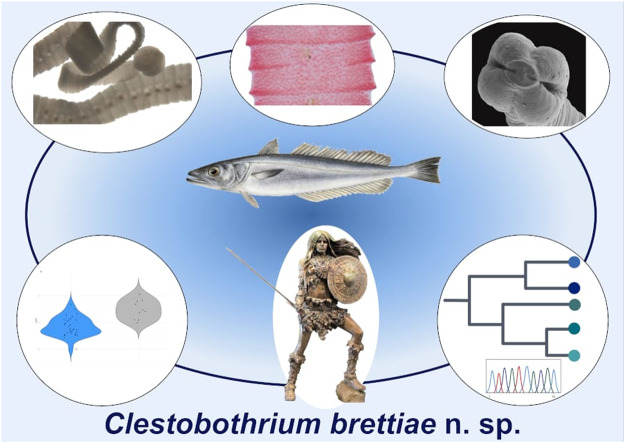

## Introduction

The order Gadiformes includes a highly diverse group of commercially important fishes, inhabiting marine, brackish, and freshwater environments. Among Gadiformes, the European hake *Merluccius merluccius* (Linnaeus, 1758) is a demersal marine species exploited by fisheries in both the North-East Atlantic (Casey and Pereiro, 1995) and Mediterranean (Oliver and Massutí, 1995; Abaunza et al., 2001). It is ranked as the third most valuable species for Mediterranean fisheries (FAO, 2020). The populations of European hake in the North-East Atlantic and the Mediterranean are well-differentiated and considered separate stocks. However, it has been suggested that distinct subpopulations of European hake exist within the Mediterranean, which can be divided into three main groups located in the Western, Central, and Eastern Mediterranean (Spedicato et al., 2022). Due to its commercial importance and intense exploitation, the helminths of the European hake have been extensively studied from both the Atlantic and Mediterranean areas. According to Gibson et al. (2005), only four cestode species use the European hake as a definitive host. These are three Bothriocephalidea [i.e. *Bothriocephalus scorpii* (Müller, 1776) Cooper, 1917, *Clestobothrium crassiceps* (Rudolphi, 1819) Lühe, 1899, and *Eubothrium rugosum* (Batsch, 1786)], and one Caryophyllidea (i.e. *Caryophyllaeus trisignatus* Molin, 1858). Within Bothriocephalidea, there are three families that include a total of ten genera with over 46 species (Kuchta et al., 2008a; Caira et al., 2025). Members of the genus *Clestobothrium* Lühe, 1899 (Bothriocephalidae), currently consist of five species that exhibit strong host specificity for Gadiformes (Kuchta et al., 2008a, 2008b; Caira et al., 2025).

During a parasitological survey of teleost fish from the Ionian Sea (Eastern Mediterranean) in southern Italy, some individuals of a bothriocephalid species were found in the intestine of European hake. These tapeworms were determined to be a morphologically distinct, previously unknown species of *Clestobothrium*. This new species is described here using an integrative taxonomic approach that combines morphological and molecular data.

## Materials and methods

### Sample collection

In December 2024, 22 individuals of the European hake were obtained from off Mirto–Crosia (39° 36′ 10·61″ N, 16° 47′ 41·14″ E; Ionian coast of Calabria Region in southern Italy), using commercial trawling operations at ∼ 400–800 m depth. Fish were refrigerated (4 °C) and transferred to the laboratory, where they were studied within 12 hr of fishing. European hakes were 15 females and seven males with total length (TL) ranging from 31·8 to 45 cm and from 28·5 to 51 cm, respectively.

During the dissection, the intestine of each fish was examined, and cestodes were obtained under a dissecting microscope (Axio Zoom V16, Zeiss, Switzerland) using the methods described in Santoro et al. (2022, 2023). Cestodes were washed in physiological saline solution, and when relaxed, they were preserved in 70% ethanol, hot formaldehyde 4%, or frozen (−20 °C) for subsequent morphological and molecular analyses.

### Morphological study

For light microscopy, cestodes were stained with Mayer’s acid carmine, dehydrated through a graded ethanol series, cleared in methyl salicylate, and mounted in permanent slides in Canada balsam (Santoro et al., 2024). The measurements (in micrometres, except where stated), reported as range values with mean ± standard deviation in parentheses, followed by the total number (*n*) of observations, were obtained using a light microscope (Axio Imager M1, Zeiss) and a dissecting microscope equipped with the ZEN 3·1 imaging system (Zeiss). Drawings were made with the aid of an XP PEN Deco 02 drawing tablet (Deco, Italy) and the software Adobe Illustrator and Adobe Photoshop.

For scanning electron microscopy (SEM) analysis, two specimens were fixed overnight in 2·5% glutaraldehyde, then transferred to 40% ethanol (10 min), rinsed in 0·1 M cacodylate buffer, postfixed in 1% OsO_4_ for 2 hr, and dehydrated in ethanol series, critical point dried, and sputter-coated with platinum. Observations were made using a JEOL JSM 6700 F scanning electron microscope operating at 5·0 kV (JEOL, Japan).

For comparative purposes the following material was borrowed and studied: syntypes (ZMB-E.1807, three slides) of *C. crassiceps* from European hake collected off Naples (Italy) (which represents the only material of *Clestobothrium* spp. available from the Mediterranean Sea) deposited in the Museum für Naturkunde, Leibniz-Institut für Evolutions und Biodiversitätsforschung, Berlin (Germany); voucher material of *C. crassiceps* (BMNH 1989.1.27.6-7; BMNH 1989.7.17.1; BMNH 1989.7.6.17; BMNH 1989.7.6.31) from the European hake collected from various locations of the Atlantic Ocean deposited in the Natural History Museum, London (England, UK).

### Biometric statistics

For morphological comparison, based on the condition of the studied material, selected biometric characters were measured from individuals of *C. crassiceps* borrowed from the Museum für Naturkunde, Leibniz-Institut für Evolutions und Biodiversitätsforschung and Natural History Museum. These values were explicitly randomised by character within *C. crassiceps* syntypes, *C. crassiceps* voucher material, and the proposed novel species, and used in a multivariate statistical approach to test biometric differences among the two species. Canonical variate analysis (CVA), using the *capscale* function from the R *vegan* package (Oksanen et al., 2024), was implemented as a constrained ordination on a Euclidean distance matrix, allowing the visualisation and quantification of group separations while accommodating multivariate data structures. The significance of the group effect was tested using permutation-based ANOVA (PERMANOVA) with 999 permutations (function *anova.cca* in the same package). All analyses were conducted in R (R Core Team, 2023).

Firstly, we tested differences in morphological characters between the proposed novel species and syntypes of *C. crassiceps* from the Mediterranean Sea (ZMB-E.1807). In this case, due to the poor conditions of the syntypes, only four morphological characters were available [i.e. immature proglottis length/width ratio (IpL_W), mature proglottis length/width ratio (MpL_W), gravid proglottis length/width ratio (GpL_W), and egg length/width ratio (EL_W)]. Then, CVA was implemented on eight characters to test differences between the proposed novel species and voucher specimens of *C. crassiceps* from the North Atlantic, borrowed from the BMNH. These characters included those mentioned before, with the addition of ovary length/width ratio (OL_W), number of testes (Tn), testes length/width ratio (TL_W), and cirrus sac length/width ratio (CSL_W). To minimise the impact of potential artefacts resulting from differences in slide preparation, staining, or mounting, given the age and condition of the specimens, all selected variables were expressed as ratios, providing a more conservative basis for comparison. For each variable included in these analyses, the number of measurements is reported in Table 1.

**Table 1.** Comparison of biometric characters between available specimens of *Clestobothrium crassiceps* and *Clestobothrium brettiae* n. sp. obtained in this study. All measurements are reported in µ (unless otherwise specified) as a range with mean ± standard deviation in brackets

Species	*C. crassiceps*	*C. crassiceps*	*C. brettiae* n. sp.
Museum collection	ZMB	BMNH	NHMUK
Location	Tyrrhenian Sea (Mediterranean)	North Atlantic	Ionian Sea (Mediterranean)
Number of specimens	2	5	6
Museum accession number	Vermes E − 1807	1989.1.27.6 − 7; 1989.7.17.1; 1989.7.6.17; 1989.7.6.31	2025.9.22.1.a-d; 2025.9.22.2.a-e; 2025.9.22.3.a-d; 2025.9.22.4.a-c
Total L (mm)	15·6 − 22·7 (19·2 ± 5·3), *n = 2*	29·2 − 33·5 (32·0 ± 2·5), *n = 3*	39·6 − 74·8 (54·4 ± 15·6), *n = 6*
Total number of proglottids	82 − 118 (100 ± 25·4), *n = 2*	104 − 182 (144 ± 39·0), *n = 3*	104 − 156 (130 ± 19·0), *n = 6*
Immature proglottids number	96, *n = 1*	57 − 182 (124 ± 63·0), *n = 3*	50 − 108 (70 ± 22·0), *n = 6*
Mature proglottids number	7, *n = 1*	7 − 30 (12 ± 15·6), *n = 3*	10 − 51 (31 ± 15·0), *n = 6*
Gravid proglottids number	7 − 15 (11 ± 5·6), *n = 2*	4 − 17 (7 ± 8·9), *n = 3*	0 − 44 (29 ± 17·0), *n = 6*
Scolex L	1133 − 1270 (1202 ± 96·8), *n = 2*	660 − 1175 (877 ± 271·7), *n = 5*	1349 − 1630 (1440 ± 98·4), *n = 6*
Scolex W	1193 − 1235 (1214 ± 29·7), *n = 2*	584 − 1228 (846 ± 300·74), *n = 5*	1225 − 1507 (1333 ± 95·8), *n = 6*
Scolex L/W ratio	0·9 − 1·0 (0·9 ± 0·1), *n = 2*	0·9 − 1·2 (1·0 ± 0·1), *n = 5*	0·9 − 1·2 (1·0 ± 0·10), *n = 6*
Bothria L	863 − 952 (894 ± 41·2), *n = 4*	501 − 1042 (701 ± 214·8), *n = 10*	1050 − 1534 (1310 ± 181·1), *n = 12*
Bothria W	370 − 480 (436 ± 58·6), *n = 3*	205 − 562 (363 ± 134·7), *n = 8*	956 − 1112 (1042 ± 69·4), *n = 12*
Bothria L/W ratio	1·8 − 2·3 (2·0 ± 0·2), *n = 3*	1·0 − 2·7 (1·9 ± 0·6), *n = 8*	1·1 − 1·4 (1·2 ± 0·1), *n = 12*
Immature proglottids L	117 − 305 (194 ± 41·1), *n = 20*	81 − 310 (150 ± 52·1), *n = 30*	143 − 474 (309 ± 83·6), *n = 30*
Immature proglottids W	396 − 682 (531 ± 70·6), *n = 20*	367 − 1725 (976 ± 410·6), *n = 30*	677 − 1865 (1343 ± 362·7), *n = 30*
Immature proglottids L/W ratio	0·2 − 0·6 (0·3 ± 0·1), *n = 20*	0·1 − 0·5 (0·2 ± 0·1), *n = 30*	0·1 − 0·6 (0·2 ± 0·1), *n = 30*
Mature proglottids L	196 − 321 (243 ± 34·3), *n = 10*	129 − 770 (315 ± 172·2), *n = 30*	341 − 766 (517 ± 111·6), *n = 30*
Mature proglottids W	966 − 1143 (1044 ± 62·2), *n = 10*	874 − 2034 (1293 ± 395·5), *n = 30*	1264 − 2028 (1674 ± 226·3), *n = 30*
Mature proglottids L/W ratio	0·2 − 0·3 (0·2 ± 0·03), *n = 10*	0·1 − 0·8 (0·3 ± 0·2), *n = 30*	0·2 − 0·6 (0·3 ± 0·1), *n = 30*
Gravid proglottids L	293 − 694 (437 ± 109·9), *n = 16*	301 − 779 (550 ± 126·9), *n = 30*	220 − 1206 (834 ± 173·7), *n = 30*
Gravid proglottids W	946 − 1324 (1107 ± 121·3), *n = 16*	951 − 2028 (1520 ± 339·9), *n = 30*	1133 − 2045 (1707 ± 187·2), *n = 30*
Gravid proglottids L/W ratio	0·2 − 0·6 (0·4 ± 0·1), *n = 16*	0·2 − 0·7 (0·4 ± 0·1), *n = 30*	0·1 − 0·7 (0·5 ± 0·1), *n = 30*
Testes number	*36 − 52	24 − 49 (35 ± 6·1), *n = 30*	28 − 60 (29 ± 18·5), *n = 30*
Testes length	*30 − 60 in diameter	27 − 88 (47 ± 15·5), *n = 30*	83 − 125 (105 ± 10·9), *n = 30*
Testes width	Not observed	30 − 77 (46 ± 10·7), *n = 30*	80 − 117 (94 ± 9·0), *n = 30*
Testes L/W ratio	Not observed	0·8 − 1·5 (1·1 ± 0·2), *n = 30*	0·8 − 1·4 (1·1 ± 0·2), *n = 30*
Cirrus sac length	53 − 109 (87 ± 16·0), *n = 12*	17 − 133 (79 ± 36·2), *n = 30*	94 − 151 (121 ± 15·5), *n = 30*
Cirrus sac width	57 − 101 (81 ± 15·6), *n = 12*	33 − 121 (81 ± 25·8), *n = 30*	88 − 155 (124 ± 19·8), *n = 30*
Cirrus sac L/W ratio	0·8 − 1·5 (1·1 ± 0·2), *n = 12*	0·5 − 1·4 (1·0 ± 0·2), *n = 30*	0·8 − 1·2 (1·0 ± 0·1), *n = 30*
Ovary length	Not observed	79 − 393 (221 ± 92·5), *n = 30*	116 − 322 (192 ± 38·8), *n = 30*
Ovary width	Not observed	320 − 562 (425 ± 76·5), *n = 30*	473 − 638 (551 ± 45·6), *n = 30*
Ovary L/W ratio	Not observed	0·2 − 1·0 (0·5 ± 0·2), *n = 30*	0·2 − 0·5 (0·3 ± 0·1), *n = 30*
Egg length	55 − 67 (61 ± 2·8), *n = 20*	54 − 72 (61 ± 3·5), *n = 30*	56 − 67 (61 ± 3·4), *n = 30*
Egg width	34 − 44 (38 ± 2·5), *n = 20*	26 − 37 (32 ± 3·4), *n = 30*	36 − 44 (40 ± 2·0), *n = 30*
Egg L/W ratio	1·4 − 1·8 (1·6 ± 0·1), *n = 20*	1·5 − 2·5 (1·9 ± 0·2), *n = 30*	1·3 − 1·7 (1·5 ± 0·1), *n = 30*

* Values reported in Gil de Pertierra et al. (2011), but not observed in the studied material.

### Molecular and phylogenetic analyses

Genomic DNA was extracted from three specimens using the Quick-gDNA Miniprep Kit (Zymo Research, USA), according to the manufacturer’s protocol. The partial Small Subunit ribosomal RNA (SSU rRNA) gene was amplified with the primers WormA and WormB (Littlewood and Olson, 2001). The PCR was performed in 25 µl reactions with 2 µl of DNA sample, 0·6 µl of each primer at 10 mM, and 10 µl of MyFi Mix (Bioline, UK), following the thermocycling conditions in Scholz et al. (2013). The D1–D3 region of the Large Subunit ribosomal RNA (LSU rRNA) was amplified using the same PCR reactions with two sets of primer pairs, allowing the amplification of two partially overlapping regions. The primer sets were ZX-1 (Scholz et al., 2013)–ECD2 (Littlewood et al., 2000), and LSU_300 F (Littlewood et al., 2000)–1500 R (Tkach et al., 2003). The thermocycling program included a preliminary denaturation step at 94 °C (3 min) followed by 40 cycles of 94 °C (30 s), 59 °C (30 s), 72 °C (2 min), and a final extension step at 72 °C (10 min). The internal transcribed spacer 2 (ITS2) gene was amplified using the primers Proteo1 and Proteo2 and the thermocycling conditions described in Škeříková et al. (2004). Amplified products were preserved at 4 °C. Amplicons were visualised in a 1% agarose gel with GelRed (Biotium, USA) stain on a ∼ 35 min, 95 V electrophoresis. Successful PCR products were purified using Agencourt AMPure XP (Beckman Coulter, USA), following the standard manufacturer-recommended protocol. Clean PCR products were Sanger sequenced from both strands using an Automated Capillary Electrophoresis Sequencer 3730 DNA analyser (Applied Biosystems, USA) and the BigDye Terminator v. 3.1 Cycle Sequencing Kit (Life Technologies, USA). The obtained contiguous sequences were assembled and edited using MEGAX v. 11 (Kumar et al., 2018). Sequence identity was verified using the Nucleotide Basic Local Alignment Search Tool (BLASTn) (Morgulis et al., 2008).

For the SSU rRNA and LSU rRNA genes, sequences of 30 species, representatives of the family Bothriocephalidae (Kuchta et al., 2008a, 2008b; Brabec et al., 2015; Santoro et al., 2024), were retrieved from GenBank (Table 2) and aligned by gene using the multiple sequence alignment package T-Coffee (Notredame et al., 2000). The alignments included the sequences generated in this study, and the outgroup *Grillotia pristiophori* (see Table 2). These were then submitted to the transitive consistency score (TCS) to verify the reliability of aligned positions and optimize the phylogenetic topology (Chang et al., 2015), and, finally, concatenated by taxon. Genetic distances among taxa for a subset of this dataset, only including the most closely related species to our specimen, were computed using the Kimura 2-Parameter (K2P) model (Kimura, 1980) with 1000 bootstrap resampling in MEGAX v. 11 (Kumar et al., 2018). According to recent studies suggesting the effectiveness of Bayesian inference (BI) in integrative taxonomic studies of parasites of fishes (Kuchta et al., 2012; Choudhury et al., 2022), the phylogenetic hypotheses in the present work were inferred using this approach implemented in MrBayes v.3.2.7 (Ronquist and Huelsenbeck, 2003). Additionally, a maximum likelihood (ML) phylogenetic tree was calculated using iQtree v. 1.6.12 (Nguyen et al., 2015), performing 5000 standard bootstrap approximations to test the phylogenetic reliability. The best-fitted evolutionary model was TIM3 + I + Γ for both SSU rRNA and LSU rRNA alignments, as suggested by jModelTest v. 2.1.10 (Darriba et al., 2012). Posterior probability distributions for the Bayesian analysis were generated using the Markov Chain Monte Carlo (MCMC) method. MCMC searches were run for 10 000 000 generations on two simultaneous runs of four chains and sampled every 1000 generations; the first 25% of samples from the MCMC algorithm was discarded as burn-in. The quality of the Bayesian analysis (parameter densities, ESS and burn-in) and the chain convergence were examined in Tracer (Rambaut et al., 2018), and trees were visualised using Figtree v. 1.4.4 (Rambaut, 2012).

**Table 2.** Information about sequences used in the phylogenetic analyses obtained from GenBank. Sequences generated in this study are shown in bold

SSU rRNA	LSU rRNA	ITS2		Geographic origin			
GenBank ID	GenBank ID	GenBank ID	Parasite species	Host species	Host family		References
PV653959	PP756387	PV662126	*Anantrum gallopintoi*	*Synodus scituliceps*	Synodontidae	Costa Rica	Santoro et al. (unpublished), Santoro et al. (2024), Santoro et al. (unpublished)
KR780966	KR780919	–	*Anantrum* sp. (PBI_609)	*Trachinocephalus myops*	Synodontidae	USA	Brabec et al. (2015)
KR780927	KR780883	–	*Anantrum tortum*	*Synodus foetens*	Synodontidae	USA	Brabec et al. (2015)
KR780941	KR780894	–	*Bothriocephalidae* gen. sp. (PBI_033)	*Epinephelus coioides*	Serranidae	Indonesia	Brabec et al. (2015)
KR780930	KR780886	–	*Bothriocephalus australis*	*Platycephalus aurimaculatus*	Platycephalidae	Australia	Brabec et al. (2015)
KR780931	KR780888	–	*Bothriocephalus carangis*	*Uraspis uraspis*	Carangidae	Indonesia	Brabec et al. (2015)
KR780968	KR780921	–	*Bothriocephalus celineae*	*Cephalopholis aurantia x spiloparaea*	Serranidae	New Caledonia	Brabec et al. (2015)
AJ228776	AF286942	AY340118	*Bothriocephalus scorpii*	*Myoxocephalus scorpius*	Cottidae	UK	Littlewood et al. (1998), Škeříková et al. (2004)
KR780952	KR780905	–	*Bothriocephalus* sp. (PBI_485)	*Micropterus dolomieu*	Centrarchidae	USA	Brabec et al. (2015)
KR780954	KR780907	–	*Bothriocephalus* sp. (PBI_525)	*Lepisosteus oculatus*	Lepisosteidae	USA	Brabec et al. (2015)
KR780929	KR780885	–	*Bothriocephalus timii*	*Cottoperca gobio*	Bovichtidae	Argentina	Brabec et al. (2015)
KR780959	KR780912	–	*Bothriocephalus travassosi*	*Anguilla marmorata*	Anguillidae	China	Brabec et al. (2015)
KR780957	KR780910	AY340114	Bothriocestus claviceps*	*Trinectes maculatus*	Achiridae	USA	Brabec et al. (2015), Škeříková et al. (2004)
KR780955	KR780908	–	Bothriocestus cuspidatus*	*Sander vitreus*	Percidae	USA	Brabec et al. (2015)
KR780953	KR780906	–	Bothriocestus kupermani*	*Lepomis gibbosus*	Centrarchidae	USA	Brabec et al. (2015)
**PV577713**	**PV090841**	**PV577095**	***Clestobothrium brettiae* n. sp.**	*Merluccius merluccius*	Merlucciidae	Italy	This study
KR780928	KR780884	AY340122	*Clestobothrium crassiceps*	*Merluccius merluccius*	Merlucciidae	UK	Brabec et al. (2015), Škeříková et al. (2004)
KR780948	KR780901	–	*Clestobothrium cristinae*	*Merluccius hubbsi*	Merlucciidae	Argentina	Brabec et al. (2015)
KR780967	KR780920	–	*Clestobothrium splendidum*	*Merluccius australis*	Merlucciidae	Argentina	Brabec et al. (2015)
KR780949	KR780902	–	*Ichthybothrium* sp. (PBI_427)	*Mesoporous crocodilus*	Distichodontidae	Sudan	Brabec et al. (2015)
KR780936	JQ811838	–	*Kirstenella gordoni*	*Heterobranchus bidorsalis*	Clariidae	Ethiopia	Brabec et al. (2015), Kuchta et al. (2012)
KR780940	KR780893	–	*Oncodiscus sauridae*	*Saurida tumbil*	Synodontidae	Indonesia	Brabec et al. (2015)
KR780939	KR780892	–	*Penetrocephalus ganapattii*	*Saurida tumbil*	Synodontidae	Indonesia	Brabec et al. (2015)
KR780934	JQ811836	–	*Polyonchobothrium polypteri*	*Polypterus senegalus*	Polypteridae	Sudan	Brabec et al. (2015), Kuchta et al. (2012)
–	–	AY340120	*Polyonchobothrium* sp.	*Mastacembelus mastacembelus*	Mastacembelidae	Iran	Škeříková et al. (2004)
DQ925317	DQ925333	–	*Ptychobothrium belones*	*Strongylura leiura*	Belonidae	Pacific Ocean	Brabec et al. (2006)
KR780932	KR780889	AY340117	*Schyzocotyle acheilognathi*	*Cyprinus carpio*	Cyprinidae	Czech Republic	Brabec et al. (2015), Škeříková et al. (2004)
KR780969	KR780922	PQ134488	*Schyzocotyle nayarensis*	*Barilius sp.*	Danionidae	India	Brabec et al. (2015), Marick et al. (2024)
KR780938	KR780891	–	*Senga lucknowensis*	*Mastacembelus armatus*	Mastacembelidae	Vietnam	Brabec et al. (2015)
KR780960	KR780913	–	*Senga magna*	*Siniperca chuatsi*	Percichthyidae	Russia	Brabec et al. (2015)
KR780937	KR780890	–	*Senga visakhapatnamensis*	*Channa punctata*	Channidae	India	Brabec et al. (2015)
KR780935	JQ811835	MW714366	*Tetracampos ciliotheca*	*Clarias gariepinus*	Clariidae	Ethiopia	Brabec et al. (2015), Kuchta et al. (2012), El-Naggar et al. (unpublished)
–	–	AF443465	*Eubothrium crassum^#^*	*Oncorhynchus mykiss*	Salmonidae	UK	Králová-Hromadová et al. (2003)
DQ642925	DQ642763	–	*Grillotia pristiophori^#^*	*Pristiophorus nudipinnis*	Pristiophoridae	Australia	Olson et al. (2010)

* Reassigned to this genus (see Caira et al., 2025). ^#^Outgroup.

For the ITS2 gene, representatives of the family Bothriocephalidae for which this gene marker was available in GenBank (ten species, including the present and the outgroup *Eubothrium crassum –* (Bloch, 1779) Nybelin, 1922, AF443465, see Table 2) were aligned as described above. Genetic distances among these taxa were estimated using MEGAX v. 11 (Kumar et al., 2018) with the Kimura 2-parameter (K2P) model (Kimura, 1980) as earlier. BI and ML phylogenetic trees were calculated according to the methodology illustrated for the SSU rRNA and LSU rRNA genes. The best-fitted evolutionary model was HKY + Γ, according to jModelTest v. 2.1.10 (Darriba et al., 2012).

## Results

**Figure 1.**
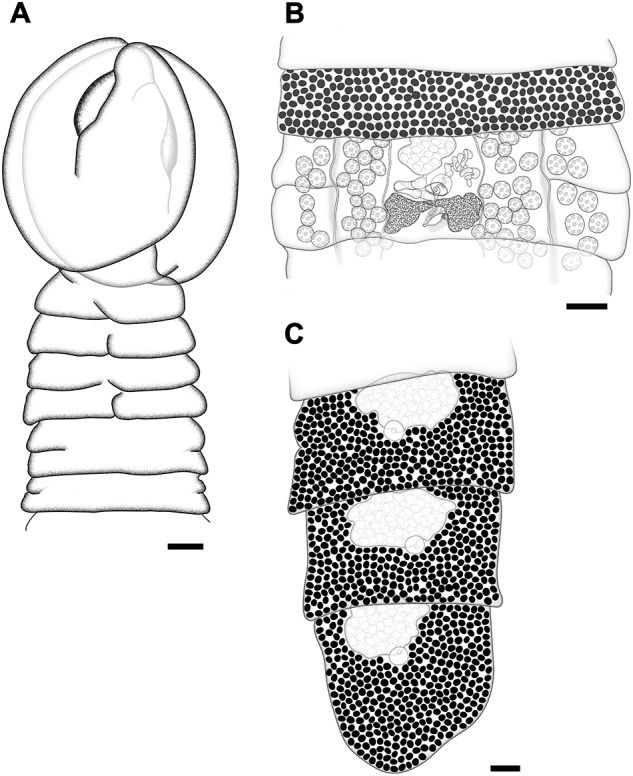
Line drawing of *Clestobothrium brettiae* n. sp. (A) Scolex of paratype (NHMUK 2025.9.22.2.a-e), (B) mature proglottis, and (C) gravid proglottids of the holotype in ventral view; scale: A–C 0·2 mm.

**Figure 2.**
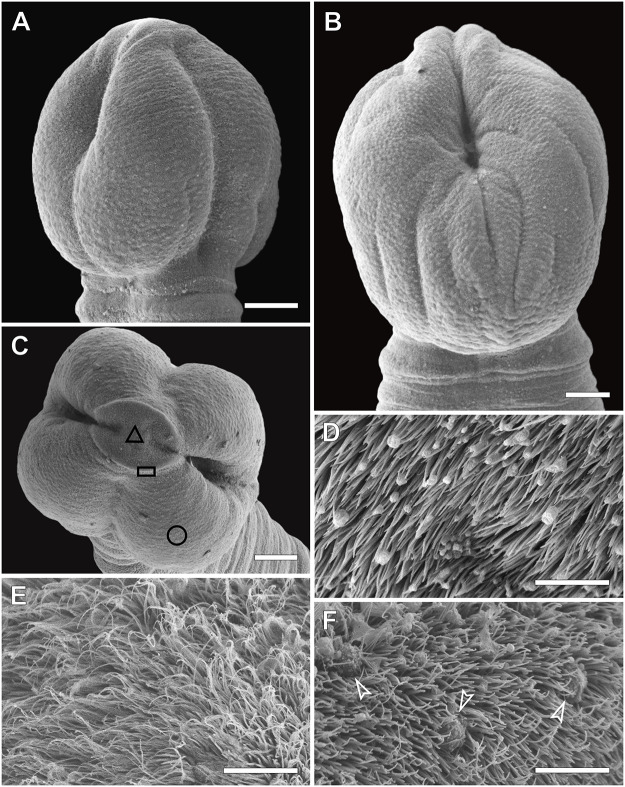
*Clestobothrium brettiae* n. sp. from the European hake, SEM micrographs of the scolex. Dorso-lateral view (A), dorso-ventral view (B), and apical view (C). Geometric figures in C indicate the surfaces shown at higher magnification showing distinct microtriches on the lateral surfaces of bothria (circle, D), on the central surface of the apical disc (triangle, E), and on the marginal surfaces of the apical disc (rectangle, F); white arrowheads indicate the tumuli. Scale: A–C 100 µm; D–F 5 µm.

**Figure 3.**
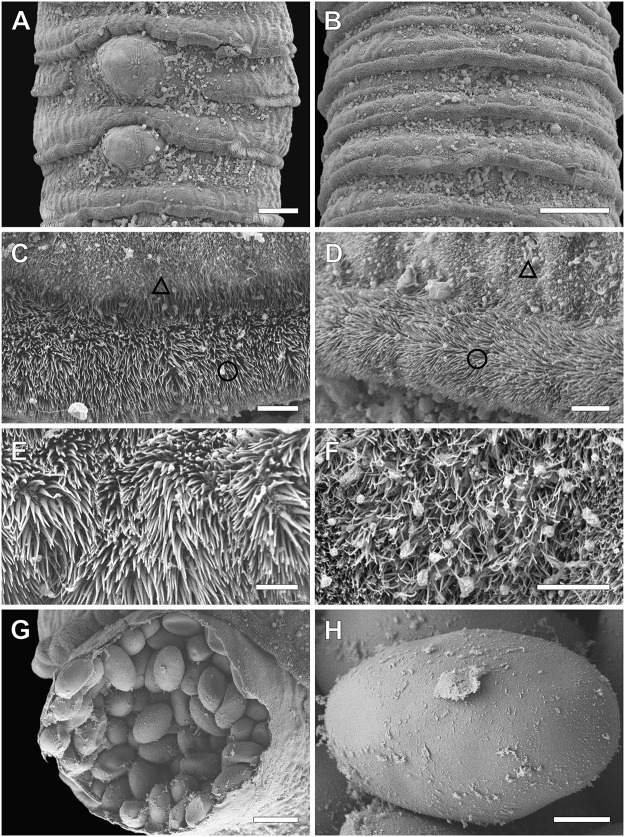
*Clestobothrium brettiae* n. sp. from the European hake, SEM micrographs of the strobila. Ventral (A) and dorsal (B) views of gravid proglottids; ventral (C) and dorsal (D) views of the posterior margin of proglottids; higher magnification of posterior margin of proglottid surfaces showing gladiate spinitriches (E) and capilliform filitriches (F), uterine sac (G) and egg (H). Geometric figures in C and D indicate surfaces shown at high magnification in E (circle) and F (triangle). Scale: A–B 100 µm; C–D 10 µm; E–F 5 µm; G 50 µm; H 10 µm.

Description (Figures 1–3)

ZooBank: LSID urn:lsid:zoobank.org:act:55593790-D2B9-48D6-B854-04660B462FA0

Taxonomic summary

Order Bothriocephalidea Kuchta, Scholz, Brabec & Bray, 2008

Family Bothriocephalidae Blanchard, 1849

*Clestobothrium brettiae* n. sp. Occhibove, López-Verdejo & Santoro, 2025

Type-host: European hake *Merluccius merluccius* (Linnaeus, 1758) (Gadiformes: Merlucciidae).

Type-locality: off Mirto-Crosia (39° 36′ 10·61″ N, 16° 47′ 41·14″ E), Ionian coast of Calabria, southern Italy, Eastern Mediterranean Sea (collected on December 11, 2024).

Location in the host: intestine.

Type-material: holotype NHMUK 2025.9.22.1.a-d and 3 paratypes NHMUK 2025.9.22.2.a-e; NHMUK 2025.9.22.3.a-d; NHMUK 2025.9.22.4.a-c deposited in the Natural History Museum, London (England, UK).

Prevalence and mean intensity: 8 of 22 fish infected (36·3%); 1·8 (range: 1–3).

Etymology: the new species is named after the legendary Amazon, Brettia, who belonged to the Brettii people. These people inhabited the northern and central areas of what is now the Calabria region in southern Italy between the 5th and 2nd centuries BC. According to legend, Brettia led a rebellion of the Brettii against the Lucanian people, who had enslaved them, guiding her people towards freedom.

Description (based on six whole mounts and two SEM preparations). All measurements and the number of measurements for each character are listed in Table 1. Medium-sized worms, flattened dorsoventrally. Body length 39·6–74·8 (54·4 ± 15·6) mm long. Strobila craspedote, anapolytic. External segmentation complete, spurious articulations (sensu Cooper, 1918) present (Figures 1A, 1B, 3A, and 3B). Proglottids wider than long (Figures 1A–C, 3A, and 3B), anterior and middle surfaces covered with capilliform filitriches (Figures 3C, 3D, and 3F), lateral and posterior surfaces covered with gladiate spinitriches along a circular cord (Figures 3C–E). Immature proglottids 143–474 (309 ± 83·6) long, 677–1865 (1343 ± 362·7) wide, length/width ratio 0·1–0·6 (0·2 ± 0·1); mature proglottids 341–766 (517 ± 111·6) long, 1264–2028 (1674 ± 226·3) wide, length/width ratio 0·2–0·6 (0·3 ± 0·1); gravid proglottids 220–1206 (834 ± 173·7) long, 1133–2045 (1707 ± 187·2) wide, length/width ratio 0·1–0·7 (0·5 ± 0·1).

Scolex globular 1349–1630 (1440 ± 98·4) long, 1225–1507 (1333 ± 95·8) wide, divided by longitudinal grooves into two dorsoventral bothria (Figures 1A and 2A–C). Bothria spherical to oval 1050–1534 (1310 ± 181·1) long, 956–1112 (1042 ± 69·4) wide.

Apical disk well developed, forming two lip-like structures perpendicular to longitudinal grooves (Figure 2C). Apical disk with tumuli (dome-shaped evaginations) (Figure 2F), covered with thick, longer capilliform filitriches on the central surface between lips (Figure 2E), and smaller filitriches on marginal surfaces (Figure 2F). Bothria covered with gladiate spinitriches (Figure 2D). Apertures of bothria deep, elongated, bordered by muscular sphincter connected anteriorly by a narrow muscle (Figures 2A–C). Neck absent. Osmoregulatory canals medullary, two pairs per each proglottis side (Figure 1B).

Testes medullary, round to oval, 28–60 (29 ± 18·5) in number per mature proglottid, 83–125 (105 ± 10·9) long, 80–117 (94 ± 9·0) wide, distributed in two lateral fields contiguous from proglottis to proglottis, not surrounding the ovary posteriorly. Genital pore round, dorsal, sub-median, posterior to posterior margin of previous proglottis. Cirrus sac round 94–151 (121 ± 15·5) long, 88–155 (124 ± 19·8) wide, thick-walled, irregularly alternating dextrally or sinistrally to median line in successive proglottis. Cirrus elongate, unarmed. Vas deferens strongly coiled, situated anterolaterally (Figure 1B).

Ovary bilobed 116–322 (192 ± 38·8) long, 473–638 (551 ± 45·6), slightly folliculate, median, curved towards posterior margin of proglottis, isthmus conspicuous. Genital atrium absent. Vaginal canal in midline of proglottis, vaginal sphincter absent. Seminal receptacle not observed. Vitelline follicles cortical, round to irregular in shape, densely distributed across entire proglottis. Vitelline reservoir overlaps ovary isthmus. Uterus tubular, irregularly alternating destrally or sinistrally to median line, forming a well-developed uterine sac (Figures 1B, 1C, 3A, and 3G). Uterine pore ventral, median. Intrauterine eggs 56–67 (61 ± 3·4) long, 36–44 (40 ± 2·0) wide, have no operculum (Figure 3H).

### Remarks

According to Caira et al. (2025) there are six species of *Clestobothrium*, including the new one. These are: *C. crassiceps* (Rudolphi, 1819) Lühe, 1899 described from off Naples (Tyrrhenian Sea, Mediterranean) from the European hake, *C. neglectum* (Lönnberg, 1893) (Dronen and Blend, 2003) described from the western coast of Sweden (Baltic Sea) from the tadpole fish *Raniceps raninus, C. gibsoni* (Dronen and Blend, 2005) described from the Gulf of Mexico (Atlantic Ocean) from the bullseye grenadier *Bathygadus macrops, C. cristinae* (Gil de Pertierra et al., 2011) from Patagonia shelf of Argentina (South Atlantic Ocean) from the Atlantic hake *Merluccius hubbsi*, and *C. splendidum* (Gil de Pertierra et al., 2011) from Patagonia shelf of Argentina from the Patagonian hake *Merluccius australis*, and finally the present novel species.

*Clestobothrium brettiae* n. sp. can be differentiated from all congeners by its unique ovary shape, and a combination of characters, such as the arrangement and number of testes. In the novel species the ovary is curved towards the posterior margin of proglottis, in contrast in all congeners it is curved towards the anterior margin of proglottis. There are 36–52 testes per proglottid in *C. crassiceps*, 70–90 in *C. neglectum*, 60–65 in *C. gibsoni*, 49–90 in *C. splendidum*, 39–64 in *C. cristinae*, and 28–60 in *Clestobothrium brettiae* n. sp. The testes are completely surrounding the ovary posteriorly (in *C. neglectum, C. gibsoni*, and *C. cristinae*) or partially (in *C. splendidum*), or not surrounding the ovary posteriorly (in *C. crassiceps* and *Clestobothrium brettiae* n. sp.).

*Clestobothrium brettiae* n. sp. is closely related to *C. crassiceps*, the only congener sharing both the absence of a genital atrium and a vaginal sphincter, along with overlapping host and geographic distribution. In addition to the ovary shape and the different number of testes, other notable qualitative differences between the novel species and *C. crassiceps* include the distribution of large gladiate spinitriches and ovary morphology (slightly folliculate *vs* markedly folliculate). In *Clestobothrium brettiae* n. sp., large gladiate spinitriches are arranged along a circular cord on the posterior surfaces of the proglottis, while in *C. crassiceps* those are arranged only on the posterior margin of the proglottis [see also redescriptions by Cooper (1918) and Gil de Pertierra et al. (2011)]. Moreover, *Clestobothrium brettiae* n. sp. has larger scolex, proglottids, testes, and cirrus sac compared to *C. crassiceps* (Table 1; see also Gil de Pertierra et al., 2011).

Evident morphological differences between the novel species and *C. gibsoni* and *C. neglectum* also include different morphology of scolex, which lacks the apical disk, a larger number of proglottids (725–770 in *C. neglectum*, 500–600 in *C. gibsoni*), and different arrangement of large gladiate spinitriches. Morphological differences between the novel species and *C. cristinae* and *C. splendidum* also include the number of osmoregulatory channels (two pairs *vs* three pairs per side), and ovary location (median *vs* equatorial).

### Biometric statistics

**Figure 4.**
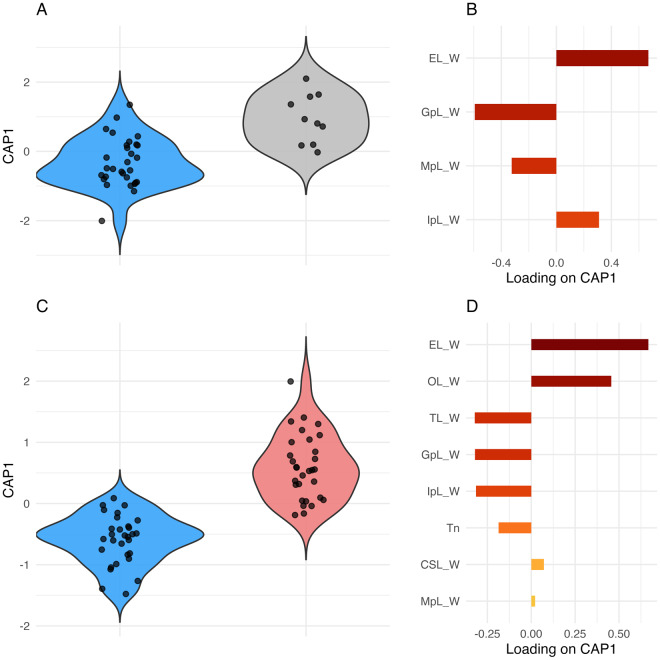
CVA results displayed as violin plots showing the distribution of scores along the CAP1 axis for each group, highlighting group-wise variation along the primary discriminant axis: *Clestobothrium brettiae* n. sp. (light blue) *vs* the syntype material of *C. crassiceps* from the Mediterranean (grey) (A); and *Clestobothrium brettiae* n. sp. (light blue) *vs* the voucher material of *C. crassiceps* from North Atlantic (coral) (B). Individual points within each violin plot represent observations (e.g. one point equals a set of single measurements of each variable) included in the analysis. Corresponding bar plots (C, D) display the relative loadings of each variable for each analysis.

Measurements of *Clestobothrium* species morphological characters investigated in the present study are listed in Table 1. In the first CVA model, *Clestobothrium brettiae* n. sp. *vs C. crassiceps* syntypes, the variation explained was 45·9%. The PERMANOVA result showed the high significance of the model (*F* = 6·69, *P* = 0·001), with the egg length/width ratio and gravid proglottis length/width ratio being the characters determining the highest variation in the data (Figures 4A and 4B). Regarding the analysis between *Clestobothrium brettiae* n. sp. and *C. crassiceps* from the North Atlantic, the CVA model explained 60·7% of the total variation and was highly significant (*F* = 11·70, *P* = 0·001) (Figure 4C). Figure 4D shows the characters mostly impacting the variability in these data. In both cases, group membership accounted for a substantial proportion of the variation in the data, namely, the effect of the species was significant, confirming that group differences were not due to chance.

### Molecular and phylogenetic analyses

**Figure 5.**
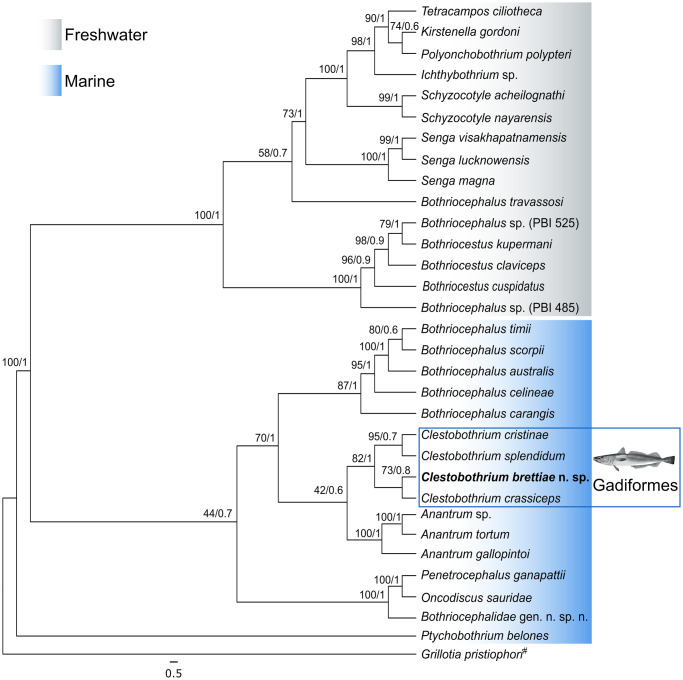
Phylogenetic tree of the representatives of the family Bothriocephalidae based on the concatenated SSU rRNA and LSU rRNA genes (alignment 3714 bp). Tree was calculated through maximum likelihood and Bayesian algorithm and shown as Bayesian tree. Bootstrap support (maximum likelihood tree) and posterior probabilities (Bayesian tree) are shown on the nodes. The scale-bar indicates the expected number of substitutions per site. The sequence generated in this study is shown in bold. The fish silhouette represents host order of genus *Clestobothrium*, while colour gradients differentiate host habitat. ^#^Outgroup.

Consensus sequences for the SSU and LSU rRNA genes were 1823 bp and 1420 bp in length, respectively, and have been deposited in GenBank under accession numbers PV577713 and PV090841. The genetic distance matrix based on the concatenated alignment revealed that the divergence between *Clestobothrium brettiae* n. sp. and its congeners exceeded the intrageneric distances observed among species of *Bothriocephalus*. In some cases, the divergence was even greater than that observed between *Bothriocephalus* species and the other three *Clestobothrium* species (Table 3). Notably, genetic distances among the well-recognised species *C. crassiceps, C. cristinae*, and *C. splendidum* were minimal (0·000–0·001), in sharp contrast to the distances between each of these and *Clestobothrium brettiae* n. sp., which ranged from 0·042 to 0·043 (Table 3). Bayesian Inference and ML phylogenetic analyses yielded identical topologies with strong nodal support, clearly resolving the genera within Bothriocephalidae and distinguishing freshwater and marine clades (Figure 5), consistent with the findings of Brabec et al. (2015). *Clestobothrium brettiae* n. sp. clustered with the other three species of *Clestobothrium*, forming a sister clade to *Anantrum*, corroborating both Brabec et al. (2015) and Santoro et al. (2024). Within the genus, *C. crassiceps* was the most closely related species, as supported by both genetic distances and morphological affinities; however, *Clestobothrium brettiae* n. sp. was clearly resolved on a distinct branch.

**Table 3.** Differences among representatives of the genera *Anantrum*, *Bothriocephalus*, and *Clestobothrium* for the concatenated SSU rRNA and LSU rRNA genes, analysed using the Kimura 2-Parameters (K2P) model (alignment length: 3714 bp). K2P value ± standard error. The sequences generated in this study are shown in bold

	Species (GenBank accession numbers: SSU rRNA, LSU rRNA)	1	2	3	4	5	6	7	8	9	10	11
1	***Clestobothriun brettiae* n. sp. (PV577713, PV090841)**	–										
2	*Anantrum gallopintoi* (PV653959, PP756387)	0·105 ± 0·009	–									
3	*Anantrum* sp. (KR780966, KR780919)	0·120 ± 0·009	0·050 ± 0·005	–								
4	*Anantrum tortum* (KR780927, KR780883)	0·118 ± 0·009	0·050 ± 0·005	0·018 ± 0·002	–							
5	*Bothriocephalus australis* (KR780930, KR780886)	0·088 ± 0·007	0·066 ± 0·007	0·077 ± 0·006	0·076 ± 0·006	–						
6	*Bothriocephalus carangis* (KR780931, KR780888)	0·074 ± 0·006	0·059 ± 0·006	0·067 ± 0·006	0·068 ± 0·006	0·033 ± 0·004	–					
7	*Bothriocephalus scorpii* (AJ228776, AF286942)	0·081 ± 0·007	0·064 ± 0·007	0·073 ± 0·006	0·075 ± 0·006	0·021 ± 0·003	0·029 ± 0·003	–				
8	*Bothriocephalus timii* (KR780929, KR780885)	0·081 ± 0·007	0·064 ± 0·007	0·071 ± 0·006	0·070 ± 0·006	0·017 ± 0·002	0·029 ± 0·003	0·013 ± 0·002	–			
9	*Clestobothrium crassiceps* (KR780928, KR780884)	0·042 ± 0·004	0·059 ± 0·006	0·065 ± 0·006	0·063 ± 0·005	0·038 ± 0·004	0·029 ± 0·003	0·033 ± 0·004	0·032 ± 0·003	–		
10	*Clestobothrium cristinae* (KR780948, KR780901)	0·043 ± 0·004	0·058 ± 0·006	0·065 ± 0·005	0·063 ± 0·005	0·038 ± 0·004	0·029 ± 0·003	0·033 ± 0·004	0·032 ± 0·003	0·001 ± 0·000	–	
11	*Clestobothrium splendidum* (KR780967, KR780920)	0·043 ± 0·004	0·058 ± 0·006	0·065 ± 0·005	0·063 ± 0·005	0·038 ± 0·004	0·029 ± 0·003	0·033 ± 0·004	0·032 ± 0·003	0·001 ± 0·000	0·000 ± 0·000	–

**Figure 6.**
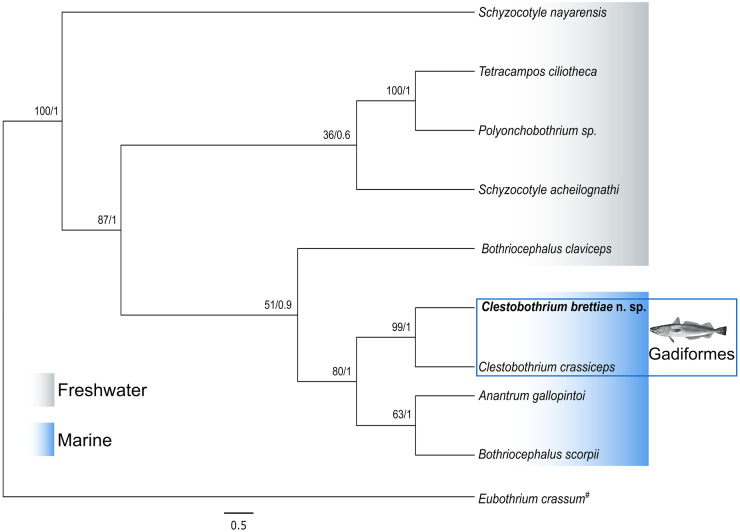
Phylogenetic tree of the available representatives of the family Bothriocephalidae based on the ITS2 rRNA gene (alignment 1041 bp). Tree was calculated through maximum likelihood and Bayesian algorithm and shown as Bayesian tree. Bootstrap support (maximum likelihood tree) and posterior probabilities (Bayesian tree) are shown on the nodes. The scale-bar indicates the expected number of substitutions per site. The sequence generated in this study is shown in bold. The fish silhouette represents host order of genus *Clestobothrium*, while colour gradients differentiate host habitat. ^#^Outgroup.

The consensus sequence of the ITS2 region was 625 bp in length (GenBank accession number PV577095). For this marker, comparative data were limited, with sequences available for only eight Bothriocephalidae species in GenBank. Nonetheless, the genetic differences between *Clestobothrium brettiae* n. sp. and the closely related *C. crassiceps* were consistent with the results obtained from other genetic markers (Table 4). Phylogenetic analyses based on the ITS2 alignment (1041 bp, including gaps) yielded congruent topologies in both BI and ML trees, with strong nodal support (Figure 6). *Clestobothrium brettiae* n. sp. was placed within the marine taxa clade, forming a well-supported, distinct branch from *C. crassiceps* (Figure 6).

**Table 4.** Differences among representatives of the family Bothriocephalidae for ITS2 rRNA sequences, analysed using the Kimura 2-Parameters (K2P) model (alignment length: 1041 bp). K2P value ± standard error. The sequence generated in this study is shown in bold

	Species (GenBank accession number)	1	2	3	4	5	6	7	8	9
1	***Clestobothriun brettiae* n. sp. (PV577095)**	–								
2	*Anantrum gallopinto*i (PV662126)	0·619 ± 0·110	–							
3	*Bothriocephalus claviceps* (AY340114)	0·208 ± 0·036	0·836 ± 0·166	–						
4	*Bothriocephalus scorpii* (AY340118)	0·166 ± 0·029	0·557 ± 0·096	0·242 ± 0·043	–					
5	*Clestobothrium crassiceps* (AY340122)	0·019 ± 0·006	0·647 ± 0·115	0·228 ± 0·040	0·190 ± 0·033	–				
6	*Polyonchobothrium* sp. (AY340120)	0·665 ± 0·118	1·866 ± 0·426	0·689 ± 0·119	0·737 ± 0·126	0·711 ± 0·129	–			
7	*Schyzocotyle acheilognathi* (AY340117)	0·500 ± 0·086	1·151 ± 0·245	0·542 ± 0·102	0·543 ± 0·096	0·556 ± 0·098	1·467 ± 0·243	–		
8	*Schyzocotyle nayarensis* (PQ134488)	0·946 ± 0·214	1·658 ± 0·409	0·747 ± 0·157	1·025 ± 0·243	1·016 ± 0·231	2·660 ± 0·598	1·555 ± 0·317	–	
9	*Tetracampos ciliotheca* (MW714366)	0·684 ± 0·166	0·848 ± 0·216	0·452 ± 0·107	0·689 ± 0·165	0·712 ± 0·171	0·129 ± 0·026	1·436 ± 0·324	0·848 ± 0·191	–

## Discussion

Diagnostic characters of genera belonging to the Bothriocephalidea were revisited and amended by Kuchta et al. (2008a). The present material well agrees with the characters of the genus *Clestobotrium* as previously described (Rees, 1958; Bray et al., 1994; Kuchta et al., 2008a). In particular, the main character used for its identification was the possession of a scolex with a sphincter surrounding the anterior aperture of bothria (Rees, 1958; Bray et al., 1994; Kuchta et al., 2008a). However, based on the most recent studies (Gil de Pertierra et al., 2011; Miquel et al., 2012; this study), we believe that a neglected character should be further added to the generic diagnosis of *Clestobothrium*. For instance, according to Kuchta et al. (2008a), the genus *Clestobothrium* is characterised by the absence of an apical disc. The presence of an apical disc has been confirmed in the present material as well as previously in its type-species *C. crassiceps* (Miquel et al., 2012), and in two other congenerics, *C. cristinae* and *C. splendidum* (Gil de Pertierra et al., 2011). Miquel et al. (2012) suggested that the presence of an apical disc on the scolex is likely a typical character for this genus; however, its presence in *C. gibsoni* and *C. neglectum* needs to be verified.

Furthermore, *C. crassiceps* was initially described with unoperculate eggs (Rudolphi, 1819; Cooper, 1918; Rees, 1958; see also Bray et al., 1994), then Rees (1958) differentiated the genus *Clestobotrium* from *Bothriocephalus* by the possession of a sphincter on the bothria and by the lack of an operculum on the eggs. The presence of an operculum on the eggs has been later confirmed in *C. crassiceps, C. gibsoni, C. cristinae*, and *C. splendidum* (Azzouz Draoui and Maamouri, 1997; Dronen and Blend, 2005; Gil de Pertierra et al., 2011; this study), but it was not observed neither in *C. neglectum* (Dronen and Blend, 2003) or in the novel species here studied using light microscopy and SEM analyses. Inside the uterus, on fixed material, and at an early stage of egg development, the operculum may not be visible yet, using light microscopy and SEM (Azzouz Draoui and Maamouri, 1997; Levron et al., 2016). In fact, Azzouz Draoui and Maamouri (1997) observed that in *C. crassiceps* the operculum appeared only after 11–13 days of development in seawater. These observations on the late differentiation of the operculum suggest that the absence of an operculum on the eggs of fixed material is not a character to use for species diagnosis.

The occurrence of microtriches on the tegumental surfaces of *Clestobothrium* spp. has been demonstrated using both light microscopy (Linton, 1901; Cooper, 1918; Dronen and Blend, 2003) and SEM analyses (Gil de Pertierra et al., 2011; Miquel et al., 2012). Linton (1901) and Cooper (1918), using light microscopy, reported that the entire scolex and the posterior margin of the proglottids of *C. crassiceps* had microtriches. The ultrastructural study of *Clestobothrium brettiae* n. sp. demonstrated the occurrence of three types of microtriches on the scolex and two distinct types on the tegument of the proglottids, showing a different distribution than that observed in *C. crassiceps*. Unfortunately, the only available SEM images of *C. crassiceps* are those of the scolex (Kuchta et al., 2008a; Miquel et al., 2012). In relaxed individuals of *C. crassiceps*, the apical disc is described as an oval structure resembling a beret, with undulating margins (Miquel et al., 2012). Miquel et al. (2012) did not mention the presence of microtriches; however, in Figures 1C and 1D of their paper, abundant large microtriches can be observed on the apical disc surface. Although the quality of those figures does not allow us to accurately characterise the microtriches, it is still possible to note that they are much larger than those observed in the present material. Moreover, the apical disc of the present material is smaller, and it does not have undulated margins, as described in *C. crassiceps* (Miquel et al., 2012) using the same fixative method.

Results of the CVA indicated statistically significant morphological differences between the newly described species and *C. crassiceps*, including both syntypes and voucher specimens. CVA, along with other multivariate methods, has been widely employed in helminth systematics to support species delimitation. These methods are particularly powerful when multiple morphometric variables are analysed, randomisation techniques are applied to ensure robustness, and significant differences are consistently observed across groups. For example, similarly to the present study, Hanzelová et al. (2005) used morphometric data and a multivariate analysis approach to analyse inter- and intra-specific variation in the genus *Eubothrium*, showing the utility of such analyses in integrative taxonomy. Ahmadi (2004) used a similar approach to analyse the morphometry of the larval rostellar hooks of *Echinococcus granulosus* to effectively distinguish among the various Iranian strains. Moreover, Hernández-Mena et al. (2014) applied this type of multivariate analysis to differentiate cryptic species of trematodes within the genus *Parastrigea*, successfully supporting molecular phylogenetic results with quantitative morphological data. These precedents, alongside the significant results in the present study, strongly support the hypothesis that the newly described specimens represent a distinct species, further validated by the number of morphometric characters examined, the use of randomised data partitioning to avoid bias, and the consistent statistical separation observed.

The high genetic divergence between *Clestobothrium brettiae* n. sp. and its congeners – particularly the genetic distance observed in the concatenated rRNA genes dataset and consistent separation in ITS2 gene analyses – far exceeded typical intrageneric variation observed in *Bothriocephalus* and even among other species of *Clestobothrium*. These genetic distances, together with congruent phylogenetic placements in both BI and ML analyses, strongly support the distinctiveness of *Clestobothrium brettiae* n. sp. as a new taxon. Phylogenetic analyses of concatenated SSU-LSU rRNA genes and ITS2 rRNA gene confirmed that, within the genus, *Clestobothrium brettiae* n. sp. and *C. crassiceps* were the most closely related species, but each was resolved on a distinct branch in both trees. This level of molecular differentiation, paired with discrete morphological characteristics and strong statistical separation in CVA, aligns with species boundaries recognised in other recent integrative taxonomic studies of parasites (e.g. Hanzelová et al., 2005; Hernández-Mena et al., 2014), reinforcing the validity of *Clestobothrium brettiae* n. sp. as a novel taxon.

The most important characters for distinguishing congenerics were the presence or absence of a genital atrium and a vaginal sphincter, as well as the arrangement and number of testes (Gil de Pertierra et al., 2011). Currently, *Clestobothrium brettiae* n. sp. and *C. crassiceps* are the only species lacking both a genital atrium and a vaginal sphincter. However, as described above, few morphological characters and biometrical differences allow the distinction between these two species. Molecular data, genetic distance, and phylogenetic analyses resolved unequivocally the morphological similarities between *Clestobothrium brettiae* n. sp. and the nominal species with available sequences in GenBank (i.e. *C. crassiceps, C. cristinae*, and *C. splendidum*), supporting the establishment of *Clestobothrium brettiae* n. sp. as a new taxon.
